# Time delays and risk factors in the management of patients with active pulmonary tuberculosis: nationwide cohort study

**DOI:** 10.1038/s41598-022-15264-w

**Published:** 2022-07-05

**Authors:** Yousang Ko, Jinsoo Min, Hyung Woo Kim, Hyeon-Kyoung Koo, Jee Youn Oh, Yun-Jeong Jeong, Hyeon Hui Kang, Ji Young Kang, Ju Sang Kim, Sung-Soon Lee, Jae Seuk Park, Yunhyung Kwon, Jiyeon Yang, Jiyeon Han, You Jin Jang

**Affiliations:** 1 Division of Pulmonary, Allergy and Critical Care Medicine, Department of Internal Medicine, Kangdong Sacred Heart Hospital, Hallym University College of Medicine, Sung-an ro 150, Kangdonggu, Seoul, 05355 Republic of Korea; 2 Division of Pulmonary and Critical Care Medicine, Department of Internal Medicine, Daejeon St. Mary’s Hospital, College of Medicine, The Catholic University of Korea, Seoul, Republic of Korea; 3 Division of Pulmonary and Critical Care Medicine, Department of Internal Medicine, Incheon St. Mary’s Hospital, College of Medicine, The Catholic University of Korea, Seoul, Republic of Korea; 4 Division of Pulmonary and Critical Care Medicine, Department of Internal Medicine, Ilsan Paik Hospital, Inje University College of Medicine, Goyang, Republic of Korea; 5 Division of Pulmonary, Allergy, and Critical Care Medicine, Department of Internal Medicine, Korea University Guro Hospital, Korea University College of Medicine, Seoul, Republic of Korea; 6 Division of Pulmonary and Critical Care Medicine, Department of Internal Medicine, Dongguk University Ilsan Hospital, Goyang, Republic of Korea; 7 Division of Pulmonary, Critical Care and Sleep Medicine, Department of Internal Medicine, Ulsan University Hospital, Ulsan University College of Medicine, Ulsan, Republic of Korea; 8 Division of Pulmonary and Critical Care Medicine, Department of Internal Medicine, Seoul St. Mary’s Hospital, College of Medicine, The Catholic University of Korea, Seoul, Republic of Korea; 9 Division of Pulmonary Medicine, Department of Internal Medicine, Dankook University College of Medicine, 119 Dandae-ro, Dongnam-gu, Cheonan, 31116 Republic of Korea; 10 Division of Tuberculosis Prevention and Control, Korea Disease Control and Prevention Agency, Cheongju, Republic of Korea

**Keywords:** Diseases, Health care, Medical research, Risk factors

## Abstract

Estimating the time delay and identifying associated factors is essential for effective tuberculosis control. We systemically analysed data obtained from the Korea Tuberculosis Cohort in 2019 by classifying delays as presentation and healthcare delays of pulmonary tuberculosis (PTB). Of 6593 patients with active PTB, presentation and healthcare delays were recorded in 4151 and 5571 patients, respectively. The median presentation delay was 16.0 (5.0–40.0) days. Multivariable logistic regression analysis showed that longer presentation delays were associated with neuropsychiatric disease [adjusted odds ratio (OR) 2.098; 95% confidence interval (CI) 1.639–2.687; *p* < 0.001] and heavy alcohol intake (adjusted OR 1.505; 95% CI 1.187–1.907; *p* < 0.001). The median healthcare delay was 5.0 (1.0–14.0) days. A longer healthcare delay was associated with malignancy (adjusted OR 1.351; 95% CI 1.069–1.709; *p* = 0.012), autoimmune disease (adjusted OR 2.445; 95% CI 1.295–4.617; *p* = 0.006), and low bacterial burden manifested as an acid-fast bacillus smear-negative and tuberculosis polymerase chain reaction-negative status (adjusted OR 1.316; 95% CI 1.104–1.569; *p* = 0.002). Active case-finding programmes need to focus on patients with heavy alcoholism or neuropsychiatric diseases. To ensure early PTB detection, healthcare providers must carefully monitor patients with malignancy, autoimmune disease, or a high index of suspicion for PTB.

## Introduction

Despite the ongoing coronavirus disease 2019 (COVID-19) pandemic, tuberculosis (TB), one of the oldest infectious diseases, remains a global public concern. In 2018, an estimated 10 million people were infected with TB worldwide^1^. In South Korea, 23,821 new cases of TB were reported in 2019, with an overall notification rate of 46.4/100,000 persons for new TB cases, accounting for a 9.9% decrease in the number of cases compared with that noted in 2018^2^. However, South Korea has a high TB incidence among high-income countries, even though the number has slowly but steadily decreased since 2012^2^. These large TB burdens and high rates are urgent public health problems and remain unexpected despite the adoption of the World Health Organization (WHO)’s End TB strategy^3^.

Early TB detection and adequate treatment are essential for reducing new transmission and TB-related mortality^3^. In turn, the time delay in TB diagnosis and treatment led to the continuous transmission of this disease in the community and aggravated the poor outcomes^4–6^. Timely diagnosis and subsequent initiation of anti-TB medications are necessary but remains challenging in several countries. However, the rates of timely and delayed diagnosis of TB and the types of treatments vary across countries depending on the country’s healthcare system, medical resources, pre-existing TB burden in the community, consideration of TB diagnosis by clinicians, and variable symptom presentation due to factors such as ageing and immunosuppression^7,8^.

Time delay in TB diagnosis is defined as the time from the initial onset of TB-related symptoms to the start of anti-TB treatment. It can be subdivided into presentation delays and healthcare system delays^9^. Previous studies have attempted to estimate the time delay in TB diagnosis and treatment and identify its risk factors^7,8,10–20^. However, these studies were conducted mainly in low-income countries with high TB burden and less frequently in high-income countries with low TB burden. Moreover, many studies have focused on only evaluating either presentation or healthcare delays in the study population. Unfortunately, these findings are not informative for South Korea, a high-income country with intermediate burden. Moreover, no nationwide epidemiological study on this topic has been conducted in Korea, and it has only been reported based on regional data^21,22^.

In this study, we aimed to assess data from the nationwide Korea TB cohort (KTBC) registry to quantify the time delay (delay due to patient presentation as well as delay related to factors specific to the healthcare system) in the clinical management of pulmonary TB (PTB) and to identify the possible risk factors associated with such delays to develop strategies to minimise such delays.

## Results

We classified 6593 patients with active PTB based on the type of delay: presentation delay and healthcare delay. Among these, we excluded patients diagnosed without symptoms, e.g., those diagnosed through a national TB contact investigation programme, an occupational or national health screening programme, and detection by chance during healthcare services of other medical problems^23,24^. We also excluded patients with unrealistic time data as they might be affected by recall bias (Fig. 2).

Table 1 describes the demographic characteristics of the enrolled patients according to each classification and shows several characteristics of PTB in South Korea. PTB was detected in 6593 patients, occurred more in men, and were common in individuals who lived alone; meanwhile, one-third of the study patients were diagnosed with PTB without any symptoms, also known as a subclinical disease. Hence, one-fourth of the total patients with PTB showed positive results on smear test, while only one-third of the patients with symptoms (clinical disease) showed positive results on smear test.

**Table 1:** Demographic, clinical, microbiologic, and radiologic data of patients with active pulmonary tuberculosis.

Variables	Overall PTB	Eligible PTB for PD	Eligible PTB for HD	Eligible PTB for OD
	(N = 6593)	(N = 4151)	(N = 5571)	(N = 3965)
Age	61.5 ± 18.9	62.8 ± 18.9	61.2 ± 18.8	62.6 ± 18.8
Sex, male	4191 (63.6)	2605 (62.8)	3528 (63.3)	2485 (62.7)
Height, cm	163.3 ± 9.9	162.9 ± 10.1	163.3 ± 10.1	162.8 ± 10.1
Body weight, kg	57.0 ± 11.4	56.1 ± 11.4	57.3 ± 11.5	56.2 ± 11.4
BMI, kg/m^2^	21.6 ± 25.8	21.6 ± 32.3	21.8 ± 30.0	21.7 ± 33.0
Area of residence, rural	1559 (23.6)	1008 (24.3)	1276 (22.9)	959 (24.2)
Economic status, poor	173 (2.1)	96 (2.3)	113 (2.0)	92 (2.3)
Employment Status, yes	1622 (24.6)	947 (22.8)	1471 (26.4)	928 (23.4)
Social status, living alone	2744 (41.6)	1787 (43.0)	2179 (39.1)	1656 (41.8)
Educational status, below compulsory education	335 (5.1)	207 (5.0)	284 (5.1)	143 (3.6)
Nationality, local	6318 (95.8)	3977 (95.8)	5349 (99.7)	3950 (99.6)
Smoking status, former or current	2865 (43.5)	1769 (42.6)	2456 (44.1)	1704 (43.0)
Alcohol intake, heavy	519 (7.9)	360 (8.7)	403 (7.2)	330 (8.3)
With symptom	4214 (63.9)	4151 (100.0)	3640 (65.3)	3965 (100.0)
Comorbidity	3922 (59.5)	2534 (61.0)	3281 (58.9)	2409 (60.8)
Prior history of TB infection	1090 (16.5)	736 (17.7)	929 (16.7)	710 (17.9)
With EPTB	524 (7.9)	425 (10.2)	458 (8.2)	418 (10.5)
Smear-positive in sputum	1735 (26.3)	1418 (34.2)	1547 (27.8)	1379 (34.8)
Suspicion for PTB in chest X-ray	4703 (71.3)	2999 (72.2)	4019 (72.2)	2881 (72.7)
Cavitation in chest X-ray	1199 (18.2)	848 (20.4)	1027 (18.4)	810 (20.4)
Bilateral disease in chest X-ray	2001 (30.4)	1463 (35.2)	1651 (29.6)	1381 (34.8)

Data are presented as mean ± standard deviation or number (%), unless otherwise stated.

*PD* presentation delay, *HD* healthcare delay, *OD* overall delay, *BMI* body mass index, *EPTB* extra-pulmonary tuberculosis, *PTB* pulmonary tuberculosis.

### Presentation delay

Figure 2 presents data on median delays, presented as the number of days, for each delay type. The median duration of presentation delay was 16 (5.0–40.0) days. Approximately three-fourths of patients with PTB visited the hospital within 40 days after the onset of symptoms. Comparison between patients with < 17 days of presentation delay and those with > 17 days are shown in Table 2. The late visit group (≥ 17 days of presentation delay) had a significantly higher proportion of patients who were former or current smokers and heavy alcoholics and those who presented with cough and/or sputum, body weight loss, and neuropsychiatric disease. In the multivariate analysis adjusted for potential confounding factors (Table 3), a longer presentation delay was associated only with neuropsychiatric disease (adjusted OR 2.098; 95% CI 1.639–2.687; *p* < 0.001) and heavy alcohol intake (adjusted OR 1.505; 95% CI 1.187–1.907; *p* < 0.001). The significant difference in symptoms such as cough and/or sputum and body weight loss may be attributed to the delay in hospital visits after the onset of symptoms and may be unrelated to the aetiology.

**Table 2:** Comparison of the characteristics of 4151 patients with PTB according to presentation delay.

	Presentation delay	Presentation delay	*p*-value
	< 17 days	≥ 17 days	
	(n = 2087)	(n = 2064)	
Age ≥ 65 years, years	1139 (54.6)	966 (46.8)	< 0.001
Sex, male, n (%)	1298 (62.2)	1207 (58.5)	0.460
Area of residence, rural	504 (24.1)	504 (24.4)	0.856
Economic status, poor	54 (2.6)	42 (2.0)	0.454
Employment status, yes	451 (21.6)	496 (24.0)	0.164
Social status, living alone	913 (43.7)	874 (42.3)	0.361
Educational status, below compulsory education	124 (5.9)	83 (4.0)	0.336
Nationality, foreign	77 (3.7)	82 (4.0)	0.686
Smoking status, former or current	823 (39.4)	946 (45.8)	< 0.001
Alcohol intake, heavy	140 (6.7)	220 (10.7)	< 0.001
Prior history of TB infection	382 (18.3)	354 (17.2)	0.330
Symptoms			
Cough and/or sputum	1162 (55.7)	1573 (76.2)	< 0.001
Dyspnea	558 (26.7)	408 (19.8)	< 0.001
Chest pain	195 (9.3)	162 (7.8)	0.086
Hemoptysis	216 (10.3)	110 (5.3)	< 0.001
Fever	479 (23.0)	276 (13.4)	< 0.001
Generalized weakness	192 (9.2)	131 (6.3)	0.001
Body weight loss	144 (6.9)	371 (18.0)	0.001
Comorbidity, present	1299 (62.2)	1235 (59.8)	0.111
Diabetes	426 (20.4)	462 (22.4)	0.130
Chronic respiratory disease	166 (8.0)	126 (6.1)	0.021
Chronic heart disease	143 (6.9)	120 (5.8)	0.181
Chronic liver disease	46 (2.2)	44 (2.1)	0.915
Chronic kidney disease	98 (4.7)	36 (1.7)	< 0.001
Neuropsychiatric disease	121 (5.8)	291 (14.1)	< 0.001
Malignancy	138 (6.6)	129 (6.3)	0.658
Autoimmune disease	20 (1.0)	29 (1.4)	0.198
Long-term use of steroid	11 (0.5)	10 (0.5)	1.000
TNF-alpha inhibitor	1 (0.0)	3 (0.1)	0.372
Gastro or jejunostomy	13 (0.6)	21 (1.0)	0.172
Organ transplantation	9 (0.4)	1 (0.0)	0.021

Data are presented as median (interquartile range) or number (%).

*TB* tuberculosis, *TNF* tumour necrosis factor.

**Table 3:** Univariate and multivariable analyses of risk factors for presentation delay.

Variable	Univariable logistic regression		Multivariable logistic regression	
	OR (95% CI)	*p*-value	adjusted OR (95% CI)	*p*-value
Age ≥ 65 years, years	0.956 (0.825–1.108)	0.550		
Smoking status, former or current	1.114 (0.962–1.289)	0.149		
Alcohol intake, heavy	1.444 (1.125–1.853)	0.004	1.505 (1.187–1.907)	0.001
Cough and/or sputum	2.091 (1.794–2.436)	< 0.001	2.127 (1.829–2.473)	< 0.001
Dyspnea	0.722 (0.610–0.856)	< 0.001	0.717 (0.608–0.845)	< 0.001
Chest pain	0.839 (0.657–1.071)	0.159		
Hemoptysis	0.437 (0.336–0.569)	< 0.001	0.441 (0.339–0.573)	< 0.001
Fever	0.489 (0.407–0.587)	< 0.001	0.492 (0.410–0.590)	< 0.001
Generalized weakness	0.693 (0.528–0.909)	0.008	0.704 (0.537–0.922)	0.011
Body weight loss	3.047 (2.435–3.813)	< 0.001	3.104 (2.486–3.877)	< 0.001
Chronic respiratory disease	0.809 (0.612–1.069)	0.136		
Chronic kidney disease	0.437 (0.280–0.682)	< 0.001	0.419 (0.270–0.650)	< 0.001
Neuropsychiatric disease	2.136 (1.659–2.750)	< 0.001	2.098 (1.639–2.687)	< 0.001
Organ transplantation	0.243 (0.030–2.001)	0.189		

*OR* odds ratio, *CI* confidence interval.

### Healthcare delay

Figure 2 presents the median healthcare delay as 5.0 (1.0–14.0) days. Similar to presentation delay, three-fourths of patients were diagnosed with PTB and treated within 14.0 days after the hospital visit. Comparison between patients with < 6 days of healthcare delay and those with > 5 days are shown in Table 4. The late diagnosis group (≥ 6 days of healthcare delay) had a significantly higher proportion of patients with malignancy, autoimmune disease, and low bacterial burden, manifested as a sputum acid-fast bacillus (AFB) smear-negative and tuberculosis polymerase chain reaction (TBPCR) smear-negative status. In the multivariate analysis adjusted for potential confounding factors (Table 5), malignancy (adjusted OR 1.351; 95% CI 1.069–1.709; *p* = 0.012), autoimmune disease (adjusted OR 2.445; 95% CI 1.295–4.617; *p* = 0.006), and low bacterial burden manifested as an AFB smear-negative and TBPCR-negative status (adjusted OR 1.316; 95% CI 1.104–1.569; *p* = 0.002) were independently associated with healthcare delay. In contrast, with symptoms, comorbidities such as neuropsychiatric disease, EPTB, high bacterial burden manifested as a sputum AFB smear-positive and TBPCR-positive status, or suspicious findings, and cavitation on chest radiography were inversely associated with early PTB diagnosis.

**Table 4:** Comparison of the characteristics of 5571 patients with PTB according to healthcare delay.

	Healthcare delay	Healthcare delay	*p*-value
	< 6 days	≥ 6 days	
	n = 2962	n = 2609	
Age > 65 years, years	1376 (46.5)	1240 (47.5)	0.435
Sex, male, n (%)	1984 (67.0)	1634 (62.6)	0.310
Prior history of TB infection	487 (16.4)	442 (16.9)	0.640
With symptom	2120 (71.6)	1520 (58.3)	< 0.001
Cough and/or sputum	1450 (49.0)	1004 (38.5)	< 0.001
Dyspnea	513 (17.3)	327 (12.5)	< 0.001
Chest pain	189 (6.4)	130 (5.0)	0.028
Hemoptysis	192 (6.5)	107 (4.1)	< 0.001
Fever	459 (15.5)	224 (8.6)	< 0.001
Generalized weakness	178 (6.0)	97 (3.7)	< 0.001
Body weight loss	317 (10.7)	140 (5.4)	< 0.001
Comorbidity	1714 (57.9)	1567 (60.1)	0.102
Diabetes	615 (20.8)	539 (20.7)	0.947
Chronic respiratory disease	172 (5.8)	176 (6.7)	0.150
Chronic heart disease	163 (5.5)	166 (6.4)	0.190
Chronic liver disease	65 (2.2)	60 (2.3)	0.856
Chronic kidney disease	78 (2.6)	87 (3.3)	0.133
Neuropsychiatric disease	295 (10.0)	198 (7.6)	0.002
Malignancy	196 (6.6)	260 (10.0)	< 0.001
Autoimmune disease	21 (0.7)	39 (1.5)	0.006
Long-term use of steroid	14 (0.5)	12 (0.5)	1.000
TNF-alpha inhibitor	2 (0.1)	2 (0.1)	1.000
Gastro or jejunostomy	23 (0.8)	30 (1.1)	0.168
Organ transplantation	8 (0.3)	7 (0.3)	1.000
With EPTB	278 (9.4)	180 (6.9)	0.001
Suggested PTB in chest X-ray	2300 (77.7)	1719 (65.9)	< 0.001
Cavitation in chest X-ray	666 (22.5)	361 (13.8)	< 0.001
Both lesion in chest X-ray	985 (33.3)	666 (25.5)	< 0.001
Microbiologic burden in PTB			
Sputum AFBS(+) with TBPCR(+)	871 (29.4)	309 (11.8)	< 0.001
Sputum AFBS(−) with TBPCR(+)	608 (20.5)	502 (19.2)	0.239
Sputum AFBS(−) with TBPCR(−)	851 (28.7)	1058 (40.6)	< 0.001

Data are presented as median (interquartile range) or number (%).

*TB* tuberculosis, *TNF* tumour necrosis factor, *EPTB* extra-pulmonary tuberculosis, *CXR* chest X-ray, *PTB* pulmonary tuberculosis, *AFBS* acid-fast bacilli smear, *TBPCR* tuberculosis polymerase chain reaction.

**Table 5:** Univariate and multivariable analyses of risk factors for healthcare delay.

Variable	Univariable logistic regression		Multivariable logistic regression	
	OR (95% CI)	*p*-value	adjusted OR (95% CI)	*p*-value
With symptom	0.626 (0.547–0.718)	< 0.001	0.625 (0.545–0.716)	< 0.001
Neuropsychiatric disease	0.754 (0.602–0.944)	0.014	0.755 (0.603–0.945)	0.014
Malignancy	1.353 (1.070–1.711)	0.012	1.351 (1.069–1.709)	0.012
Autoimmune disease	2.455 (1.300–4.637)	0.006	2.445 (1.295–4.617)	0.006
With EPTB	0.630 (0.500–0.794)	< 0.001	0.633 (0.502–0.797)	0.006
Suggested PTB in chest X-ray	0.599 (0.512–0.700)	< 0.001	0.598 (0.512–0.699)	< 0.001
Cavitation in chest X-ray	0.740 (0.629–0.870)	< 0.001	0.737 (0.627–0.867)	< 0.001
Both lesion in chest X-ray	0.861 (0.755–0.983)	0.026		
Sputum AFBS(+) with TBPCR(+)	0.404 (0.338–0.483)	< 0.001	0.390 (0.330–0.461)	< 0.001
Sputum AFBS(−) with TBPCR(−)	1.320 (1.107–1.574)	< 0.001	1.316 (1.104–1.569)	0.002

*OR* odds ratio, *CI* confidence interval, *PTB* pulmonary tuberculosis, *EPTB* extra-pulmonary tuberculosis, *CXR* chest X-ray, *AFBS* acid-fast bacilli smear, *TBPCR* tuberculosis polymerase chain reaction.

## Discussion

This study is the first to investigate the possible types and causes of delay and the elapsed time between symptom onset and treatment initiation in South Korea using a large sample of the KTBC database. We found significant epidemiological evidence supporting the effective control of PTB.

First, the median duration of the presentation delay was 16.0 days; this result could be disappointing as WHO and the national active case-finding programme recommend PTB screening for individuals who have been experiencing cough for two weeks or more. However, two-thirds of the patients visited the hospital within 40 days after the onset of symptoms, while one-half of the patients visited the hospital within 16 days. This finding shows that although the diagnosis was not too delayed in the majority of patients with PTB, the excessive delay in some patients had a great impact on the mean duration of delay. After adjusting for potential confounding factors, the longer presentation delay was affected by heavy alcohol intake and underlying conditions, such as neuropsychiatric disease. This finding suggests that future programmes for active case finding will need to focus on vulnerable patients with neuropsychiatric disease, those who are heavy alcoholics, and those who are unlikely to note or seek help for their symptoms.

It is very important to identify the risk factors that contribute to presentation delay as these will serve as the targets of active case-finding programmes. However, presentation delay and its associated risk factors vary greatly depending on the situation of each country. Widely different results have been reported in previous studies. In high-income countries, older age and language barriers were frequently associated with prolonged delays in one country but were regarded as unrelated factors in other countries^8,11,12^. In contrast, low income (uninsured), low educational level, smoking, old age, and female sex were frequently associated with prolonged delays in two countries, whereas male sex was found to have greater association with prolonged delays in other countries. On the other hand, in our analysis of KTBC 2019, it did not show differences between male and female gender in not presentation delay but also healthcare and overall delay. In addition, previous studies reported the lack of knowledge regarding TB, human immunodeficiency virus (HIV) infection, and immunosuppressive therapy as risk factors for prolonged delays in low and middle-income countries^10,15,16,19,25,26^. Thus, the risk factors vary among countries depending on the cultural characteristics, socioeconomic status, and the healthcare system. Thus, delays cannot be attributed to only one cause; hence, problems must be viewed from multiple perspectives and their connection should be determined in order to resolve them.

Second, the median healthcare delay was only 5.0 days. This result is similar to that of the presentation delay. A few have determined the overall trend. After adjusting for potential confounding factors, the longer healthcare delay was affected by malignancy, autoimmune disease, and low bacterial burden manifested as an AFB smear-negative and TBPCR-negative status. According to previous studies, the risk factors of healthcare delay are also diverse, similar to presentation delay, but more concise. They can be broadly divided into two categories, mostly related to the healthcare resources and system-associated diagnostics, such as referral to TB specialists and limited laboratory tests^8,11,15,16,18,22^. In the present study, the healthcare delay was also similar to that found in previous studies, which have reported that healthcare delays are related to the underlying host conditions and to disease characteristics, such as old age, immunosuppression cause, and low microbiologic burden^7,12,22,27^.

The risk factors of healthcare delay identified in our study were not much different to those previously reported, whereas those of presentation delay considerably varies from those reported in previous studies. The characteristics of patients with TB have been changing especially in high income countries including South Korea, with becoming an aging society. TB patients expected to have another medical illness before an aging society. In these changings of epidemiological condition, special attention and understanding of the risk factors of healthcare delay will improve TB control without delay of diagnosis^28^. To improve the healthcare delay according to our data, cases with malignancy and autoimmune disease as comorbidity should be focused and evaluated carefully for possibility of TB infection. TB does not have unique clinical feature and can occur in any site in human body, then it could be easily misdiagnosed as other disease and finally resulted in diagnostic delay^29,30^. In another important axis of healthcare delay, early form of PTB with low bacterial burden should also be diagnosed in a timely manner. In our data, AFB smear-negative group has less cavitation and bilateral disease on their chest X-ray. Moreover, they have less symptoms including cough/sputum, generalized weakness and loss of body weight except chest pain. These characteristics are still acting as hurdle that make early diagnosis of PTB difficult despite advances in medical diagnostic technology. It could be a time-limiting step as it is difficult to consider several factors such as TB incidence in the community, individual characteristics, medical resources, referral system, and clinical suspicion of healthcare providers. However, this measure should be performed to eliminate or at least control the TB infection.

This study has several limitations. First, this study was conducted in South Korea, a high-income country with an ageing population, an intermediate TB burden, and a low prevalence of HIV. Thus, this limitation could overestimate or underestimate our findings. Second, this study was conducted using a questionnaire, which included questions on the date of symptom onset. This means that recall bias may have influenced the presentation delay on the patient side. Moreover, on the researcher side, confirmation bias (the tendency to favour PTB-related symptoms and to neglect non-specific symptoms of PTB) may have influenced the presentation delay when we assessed the duration of patient-reported symptoms through interviews after PTB diagnosis.

Third, the study results were based on PTB cases in PPM participating hospitals. In South Korea, more than 70% of all TB patients were diagnosed and treated in PPM hospitals. Most PPM hospitals are university-affiliated hospitals. Thus, patients with severe and complicated diseases were over-represented in our study. This feature could overestimate our findings and lead to a selection bias.

Fourth, as just mentioned before, this study was conducted in high-income and using by data of PPM participating hospitals consisted of advanced medical institution based on national TB elimination project of South Korea. In these clinical conditions, we could not investigate the role of Xpert assay, a rapid and automated real-time PCR, in the early diagnosis of PTB, because it had been already widely used since 2014 in South Korea and, also in almost hospitals of this study. Thus, we could not collect Xpert results different from other TBPCR results. This factor may have influenced the healthcare delay. However, we believe that the rapid TBPCR helped to improve the early diagnosis of TB compared to before introduction of it although the nationwide data is lacking^31,32^.

Fifth, the time data of the presentation delay may differ depending on passive case-finding and active case-finding scenarios, given that its definition is the time from symptom onset to hospital visit for the work-up of symptoms and from symptom onset to hospital visit for screening. Thus, the presentation delay for active case-finding may be shorter than that for passive case-finding. However, we hypothesised that presentation delay would occur only in symptomatic patients, given that South Korea has a nationwide health insurance system, and it permits functioning of all citizens medical services regardless of economic problems. Korean individuals are more likely to visit medical facilities than those from other Organization for Economic Co-operation and Development countries, indicating easy accessibility to medical services. We assumed that symptomatic patients would have visited a hospital. However, in the real world, some patients may not have visited a medical institution for individual reasons despite the presence of symptoms. For these reasons, the KTBC has decided to investigate the reasons for hospital visits to understand diagnostic situations in detail, beginning in March 2022, through a meeting with the K-CDA.

In conclusion, we measured the durations of presentation and healthcare delays (median: 16 days and 5 days, respectively). After assessing the risk factors, we found that different factors were more associated with presentation delay compared with healthcare delay. This might be caused by the different clinical situations of the delay type. Our findings suggest that active case-finding programmes need to focus on patients with heavy alcohol intake or neuropsychiatric diseases to reduce the transmission of TB in the community. Moreover, healthcare providers should pay more attention to patients with malignancy or autoimmune disease and those with a high index of suspicion for PTB to diagnose the disease early.

## Methods

### The public–private mix project and KTBC database

In South Korea, the Korea Disease Control and Prevention Agency (K-CDA) is the governing organization of the National TB Program based on the TB Prevention Act^33^. K-CDA has been managing all TB control programmes in both the public and private health sectors. However, the healthcare service in South Korea is privatised, supported by the single-payer National Health Insurance system. The majority of TB patients were treated at private institutions (80.3% in 2009) and the proportion of these patients has increased to 96.9% in 2019^2^. Therefore, the K-CDA has made it mandatory to report TB patients being managed at private institutions. Moreover, the K-CDA established a TB monitoring system using the Korean National TB Surveillance System, a web-based system launched in 2000. For these reasons, the national public–private mix (PPM) PPM TB control project was started in 2009 and has gradually expanded^34,35^. It is operated by the Korean Academy of Tuberculosis and Respiratory Diseases under the supervision of K-CDA. However, it had innate limitations of a surveillance programme and comprised only 25 monitoring indicators, despite covering most of the nation’s TB programme^34^. Thus, it is necessary to further investigate in more detail the characteristics of TB cases, with the aim of an advanced National TB Elimination Project.

For these reasons, KTBC was planned to further improve TB control. It is a nationwide, prospective, and observational cohort comprising active TB cases from 172 hospitals in 21 districts (PPM participating hospitals) in South Korea. This study aimed to identify the epidemiological characteristics and observe the clinical outcomes of patients with active TB since July 2018. Based on the National TB Program, each TB patient was notified and followed up with the TB case report form. Since then, all notified TB cases from each hospital were consecutively enrolled. In addition to the report, the KTBC included responses to in-depth detailed interviews and investigations, including information about comorbidities, height, body weight, economic status, employment status, social status, education level, and symptoms as well as information about the programme, including initiation, discontinuation, termination, adverse effects, and mortality. Each TB case was followed up every month during the course of anti-TB treatment, based on the recommendations of the K-CDA. TB specialist nurses of each hospital interviewed the patients and filled out the case-level forms. The collected data were checked by regional and central data managers. After a regional and central audit, the data were analysed and organised by a central statistical team.

**Figure 1:**
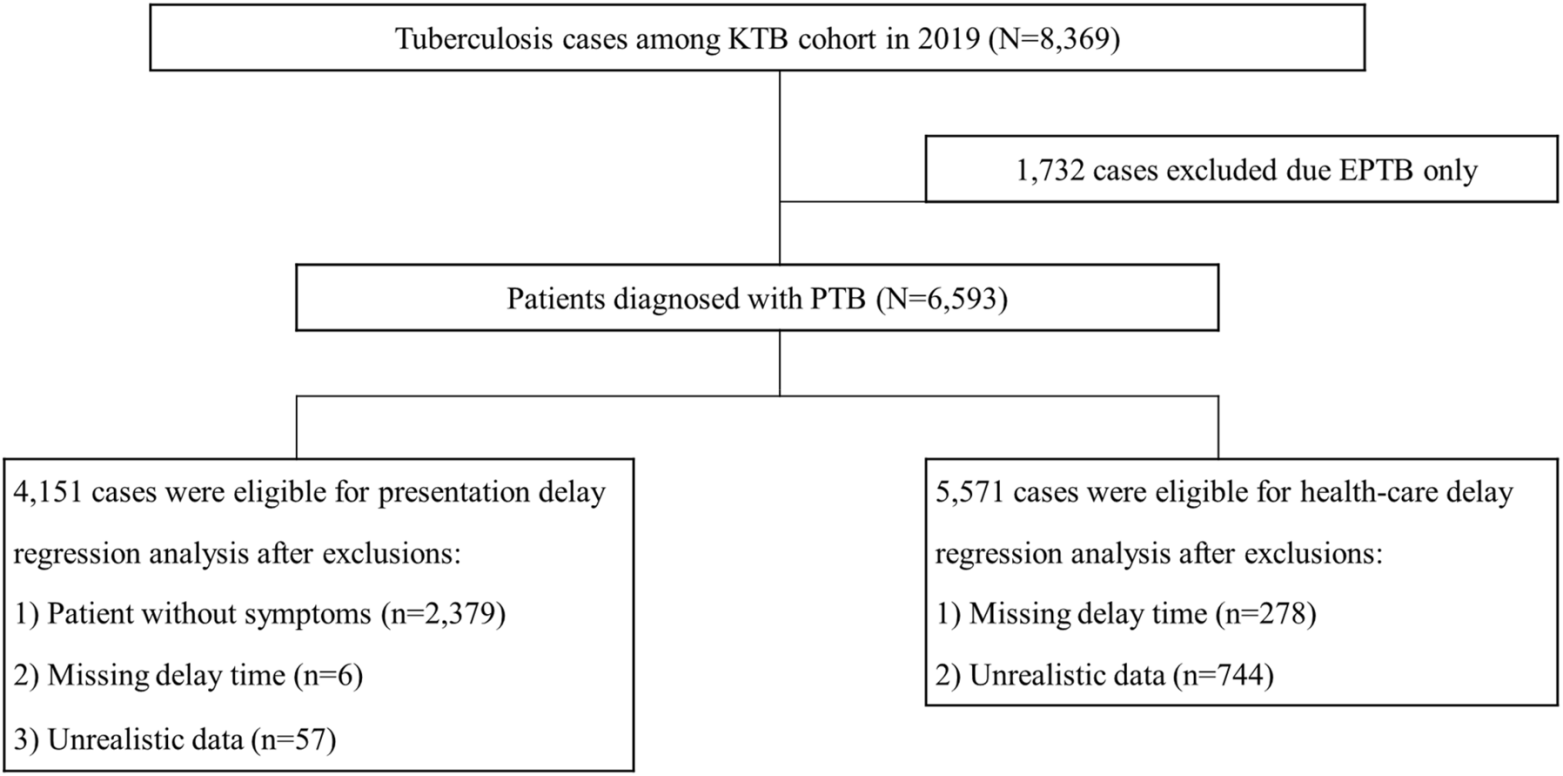
Flow diagram of the study. Among the patients with TB in the KTBC in 2019, those with EPTB without PTB were excluded. Patients diagnosed with PTB initially, but not true, were also excluded. Then, we classified the patients with PTB according to each study’s aim. First, to investigate presentation time delay and risk factors, patients without symptoms, with missing data, or with unrealistic time variables were excluded from the analysis. Second, to investigate healthcare time delay and risk factors, patients with missing data or unrealistic time variables were also excluded. Unrealistic time delay was defined as < 1 day of presentation and healthcare delay. Patients without symptom were identified as result of contact with a TB patient through the occupational and national health screening programme. Cases with unrealistic data were defined as the calculated delay was < 1 day. *KTBC* Korea Tuberculosis Cohort, *EPTB* extra-pulmonary tuberculosis, *PTB* pulmonary tuberculosis.

To achieve the research purpose, only PTB cases were included as these patients represent the index cases for TB transmission in the community. Then, we classified cases with PTB according to each study’s aim (Fig. 1). In 2019, 8325 TB patients were registered in the KTBC database. Of these, 1776 (21.3%) were excluded due to extra-PTB (EPTB). Finally, the study enrolled 6593 patients with active PTB.

### Definitions of variables

PTB was defined as TB involving the lung parenchyma. EPTB was defined as TB involving organs other than the lungs; this included the pleura without radiographic abnormalities in the lungs, lymph nodes, abdomen, genitourinary tract, skin, joints and bones, and meninges, with the confirmed presence of at least one *Mycobacterium tuberculosis* specimen. A patient with both PTB and EPTB TB was classified as PTB^36^.

**Figure 2:**
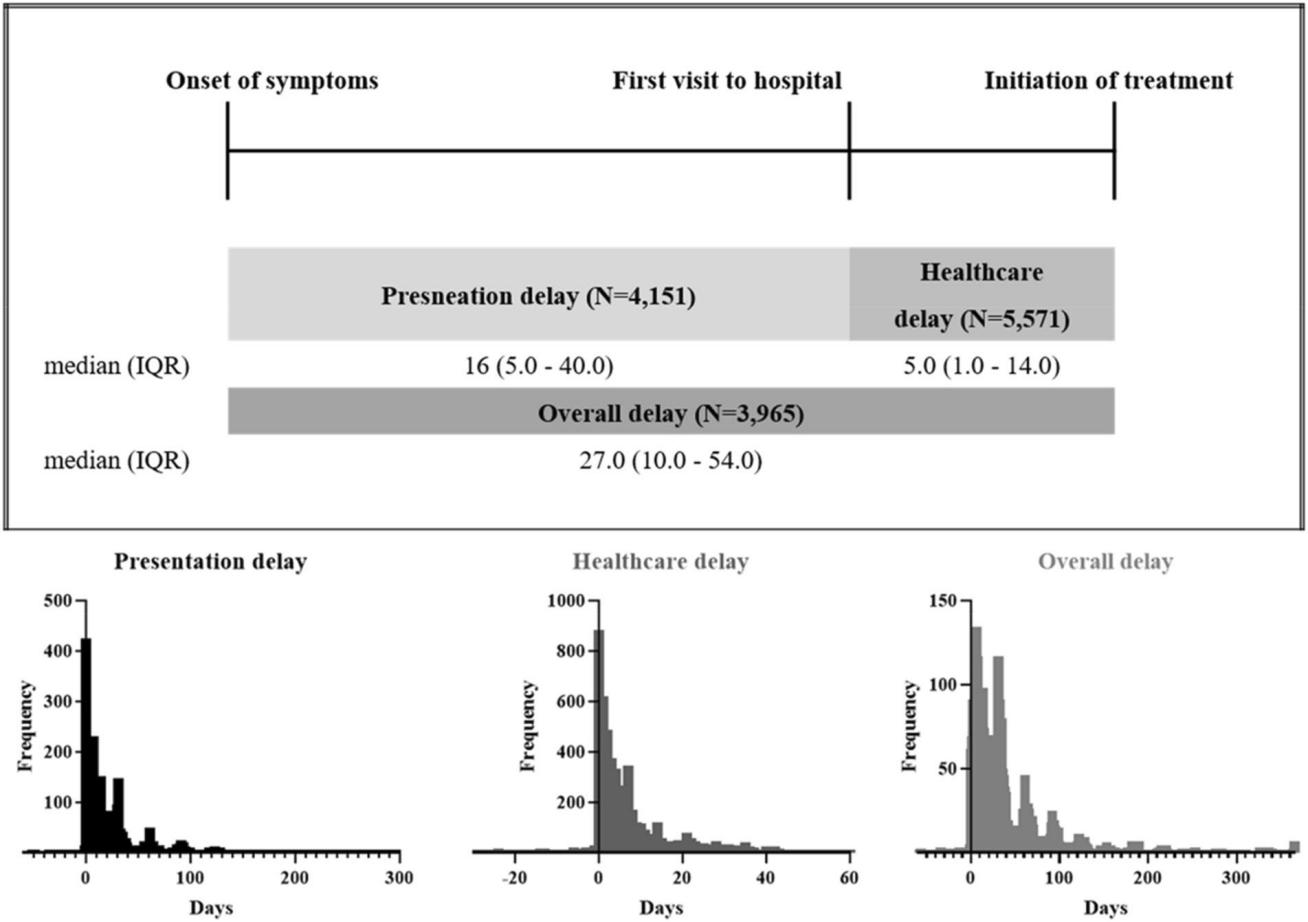
Definitions of each delay during the pulmonary tuberculosis diagnosis period. The overall delay was also measured. The median overall delay from the development of symptoms to the initiation of treatment for PTB was 27.0 (10.0–54.0) days. These parameters were estimated in 3965 patients using both presentation and healthcare delay data. We did not investigate the risk factors because of the complexity and heterogeneity of each delay. The histogram at the bottom of figure shows the frequency distribution for each time delay. *IQR* interquartile range.

We divided the time interval of PTB into presentation and healthcare delays^9^. A presentation delay was defined as the duration between PTB-related symptom onset and the first hospital visit. Healthcare delay was defined as the duration between the first hospital visit and initiation of anti-PTB treatment after diagnosis of PTB infection (Fig. 2).

### Ethics statement

The Institutional Review Board of Hallym University Kangdong Sacred Heart Hospital approved the study protocol and waived the need to obtain informed consent from the patients because the study was purely observational, with analysis of anonymised patient data, without interventions. The K-CDA has the authority to hold and analyse surveillance data for public health and research purposes. All methods were performed in accordance with the relevant guidelines and regulations.

### Statistical analysis

Data are presented as medians and interquartile ranges (IQRs) for continuous variables and as numbers (percentages) for categorical variables. Data were compared using the Mann–Whitney U test for continuous variables and Fisher’s exact test for categorical variables. The time data of presentation and healthcare delays showed a non-normal distribution. Therefore, a multiple logistic regression analysis was used to identify independent risk factors of each presentation and healthcare delay, as measured by the estimated odds ratios (OR) with 95% confidence intervals (CIs), including variables with a *p*-value of < 0.1 on univariate analysis^37^. There is no established cut-off for the presentation and healthcare delays because of the differences in variables across countries. We used the operational definitions of presentation and healthcare delays according to the calculated median times among the eligible PTB patients, which was 16 and 5 days, respectively. We performed a binary logistic regression analysis using the two groups divided based on the median number of days for each delay. To reduce the risk of multi-collinearity, one closely correlated variable was a candidate for inclusion in the final model. All the tests were two-sided, and a *p*-value of < 0.05 was considered to indicate statistical significance. Data were analysed using IBM SPSS Statistics version 26 (IBM Corp., Armonk, NY, USA) and Graph Pad Prism 9.0 (Graph Pad, San Diego, CA, USA).

## Data Availability

Data cannot be shared publicly because of the data sharing policies at the Korea Centers for Disease Control and Prevention Agency. It is only available for researchers who meet the criteria for access to confidential data, subject to approval from respective ethics review committee at the Korean Academy of Tuberculosis and Respiratory Diseases.

## Abbreviations

COVID-19: Coronavirus disease 2019
TB: Tuberculosis
WHO: World Health Organization
KTBC: Korea tuberculosis cohort
PTB: Pulmonary tuberculosis
K-CDA: Korea Disease Control and Prevention Agency
PPM: Public-private mix
EPTB: Extrapulmonary tuberculosis
IQR: Interquartile range
OR: Odds ratios
CI: Confidence interval
HIV: Human immunodeficiency virus
